# Does arterial hypertension influence the onset of Huntington's disease?

**DOI:** 10.1371/journal.pone.0197975

**Published:** 2018-05-23

**Authors:** Leire Valcárcel-Ocete, Asier Fullaondo, Gorka Alkorta-Aranburu, María García-Barcina, Raymund A. C. Roos, Lena E. Hjermind, Carsten Saft, Marina Frontali, Ralf Reilmann, Hugh Rickards, The REGISTRY investigators of the European Huntington’s Disease Network (EHDN), Ana M. Zubiaga, Ana Aguirre

**Affiliations:** 1 Department of Genetics, Physical Anthropology and Animal Physiology, University of the Basque Country (UPV/EHU), Leioa, Spain; 2 Human Genetics, University of Navarra, Navarra, Spain; 3 Genetics Unit, Basurto University Hospital, OSI Bilbao Basurto, Bilbao, Spain; 4 Department of Neurology, Leiden University Medical Centre (LUMC), Leiden, The Netherlands; 5 Danish Dementia Research Centre, Neurogenetics Clinic, Department of Neurology, Rigshospitalet, University of Copenhagen, Copenhagen, Denmark; 6 Huntington-Zentrum (NRW) Bochum, St. Josef-Hospital, Bochum, Germany; 7 Institute of Experimental Medicine, CNR, Rome, Italy; 8 George-Huntington Institute and Institute for Clinical Radiology, University of Muenster, and Dept. of Neurodegenerative Diseases and Hertie Institute of Clinical Brain Research, University of Tuebingen, Germany; 9 Department of Neurology, University of Birmingham, Birmingham, United Kingdom; Centre de Recherche Jean-Pierre Aubert, FRANCE

## Abstract

Huntington’s disease (HD) age of onset (AO) is mainly determined by the length of the CAG repeat expansion in the *huntingtin* gene. The remaining AO variability has been attributed to other little-known factors. A factor that has been associated with other neurodegenerative diseases is arterial hypertension (AHT). The aim of this study is to evaluate the contribution of AHT to the AO of HD. We used data from a cohort of 630 European HD patients with adult onset collected by the REGISTRY project of the European Huntington’s Disease Network. Multiple linear regression and ANOVA, controlling for the CAG repeat number of the expanded allele (CAGexp) of each patient, were performed to assess the association between the AHT condition and the AO of the motor symptoms (mAO). The results showed a significant association between AHT and mAO, especially when we only considered the patients diagnosed with AHT prior to manifesting any HD signs (pre-HD AHT). Remarkably, despite the low number of cases, those patients developed motor symptoms 5–8 years later than normotensive patients in the most frequent CAGexp range (40–44). AHT is an age-related condition and consequently, the age of the patient at the time of data collection could be a confounder variable. However, given that most pre-HD AHT patients included in our study had started treatment with antihypertensive drugs prior to the onset of HD, and that antihypertensive drugs have been suggested to confer a neuroprotective effect in other neurodegenerative diseases, raises the interest in elucidating the impact of AHT and/or AHT treatment in HD age of onset in further studies. A confirmation of our results in a larger sample set would open the possibility to significantly improve HD management.

## Introduction

Huntington’s disease (OMIM: 143100) AO is mainly (about 60%) determined by the length of the CAG repeat expansion (CAGexp) in the *HTT* gene. The remaining variability has been attributed to genetic and little-explored environmental factors [1]. Arterial Hypertension (AHT) is a risk factor for numerous diseases with controversial effect on neurodegenerative diseases [2]. The impact of AHT on HD has not been examined to date. The aim of this study was to explore whether AHT could be an AO modifier factor for HD.

## Methods

European Huntington’s Disease Network’s (EHDN) REGISTRY project provided data on 1,011 HD individuals. For this study, we gathered information on mAO [3], sex and age at the time of data collection of 630 European adult-onset HD patients with CAGexp ranging between 40 and 50, with known AHT status (86 hypertensives *vs*. 544 normotensives) and with AHT onset date available in the REGISTRY dataset. The 40–50 CAGexp range was selected to avoid the inclusion of juvenile-onset HD cases and thus, the introduction of errors in the regression analysis [3–5]. Furthermore, 40–50 CAGexp was the repeat range of hypertensive patients in our sample. Consequently, comparisons were performed between hypertensives and normotensives exhibiting equivalent CAGexp distribution. Ethical approval and written informed consents for each participant were obtained by the EHDN from the local ethics committees (http://www.euro-hd.net/html/registry), in compliance with the Declaration of Helsinki, the International Conference on Harmonisation—Good Clinical Practice (CH-GCP), and local regulations. Specific ethical approval of the study was obtained from the Clinical Research Ethics Committee of the Basque Country (CEIC- Euskadi), the Human Research Ethics Committee of the University of the Basque Country (CEISH) and the EHDN Scientific Bioethical Advisory Committee (SBAC). Multiple linear regression and ANCOVA tests were used to analyze the relationship between mAO and AHT, using CAGexp value as a covariate in each regression. Kolmogorov-Smirnov (K-S) test was used to compare CAGexp and mAO distributions between normotensive and hypertensive patients, while Mann-Whitney *U* test was used, in the same way, to compare the medians(SPSS ver.23.0; SPSS Inc.).

## Results

In patients with CAGexp ranging between 40 and 50, AHT explains HD mAO variability significantly (regression analysis P = 0.016, Table 1; ANCOVA test P = 0.004). Remarkably, mAO in HD patients with AHT was manifested on average 7 years later than in normotensives (52.5 *vs*. 45 years, P<0.0001), suggesting that AHT is associated with the appearance of motor symptoms at a later age. A significant association between AHT and a higher mAO was still detected in patients who manifested AHT before HD symptoms (pre-HD AHT patients) (P = 0.024, Table 1). Despite the limited number of patients (N = 28), we detected that the mAO median value was 10 years higher in pre-HD AHT patients than in normotensive patients.

**Table 1:** Multiple regression analyses and descriptive statistics for mAO.

Model	N	Adjusted R^2^	P-value	Groups	N	Motor AO		
						Median	Mann-Whitney *U* test (P)	Kolmogorov-Smirnov test (P)
*HTT* CAGexp + AHT (40–50 CAGexp range)	630	0.592	0.016	With AHT	86	52.5	<0.0001	<0.0001
				Normotensive	544	45		
*HTT* CAGexp + Pre-HD AHT (40–50 CAGexp range)	572	0.604	0.024	Pre-HD AHT	28	55.5	<0.0001	<0.0001
				Normotensive	544	45		
*HTT* CAGexp + Pre-HD AHT (40–44 CAGexp range)	384	0.340	0.020	Pre-HD AHT	27	56	0.002	0.011
				Normotensive	357	50		

**Fig 1:**
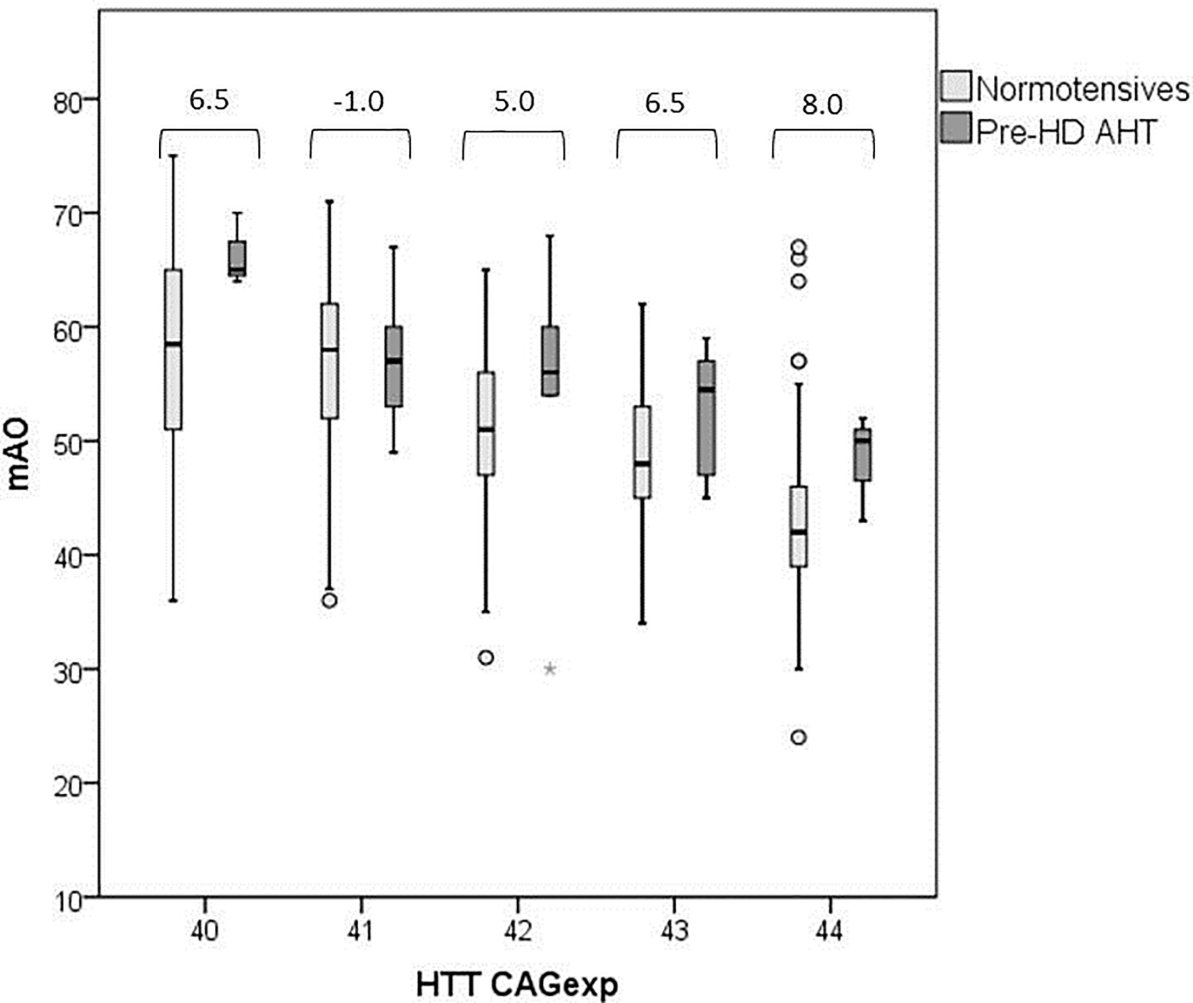
Variance in mAO for each CAGexp allele (40–44 range) in normotensives and in pre-HD AHT patients. Numbers above the square brackets are the difference in median years between Pre-HD AHT and normotensives for each CAGexp allele.

Differences in CAGexp distribution among pre-HD AHT and normotensive patients were detected (K-S test P<0.0001), probably due to sample size differences. Thus, we focused on HD patients carrying the most frequent CAGexp alleles (ranging from 40 to 44). In this case, the CAGexp allele distribution did not now differ between hypertensives and normotensives (P = 0.266, K-S test). However, the association between AHT and mAO (P = 0.020, Table 1) and the differences in the mAO distribution (P = 0.011, K-S test) remained significant. Remarkably, pre-HD AHT developed motor symptoms 6 years later than normotensives on average (56 *vs*. 50 years, P = 0.002). Interestingly, the tendency of pre-HD AHT patients to manifest HD at higher mAOs relative to normotensives was detected for each CAGexp repeat number, except for the 41 CAGexp allele and the median difference ranged between 5 and 8 years (Fig 1).

## Discussion

AHT, the most prevalent cardiovascular disorder in developed countries, has been associated with neuronal disorders such as Parkinson’disease (PD) and Alzheimer’s disease (AD) [6–8], suggesting that it may affect the neurodegeneration process. Our study is the first exploring the impact of AHT on HD and the first revealing an association between AHT and mAO.

According to our results, patients with AHT show the HD symptoms later (median of 7.5 years) than normotensive patients. These differences are more evident (median of 10.5 years) in the analysis that only takes into account patients that exhibited AHT before HD onset (pre-HD AHT patients). The results of the pre-HD AHT individuals are particularly relevant, since having manifested AHT before HD onset is a requeriment for the examined variable to be considered a mAO modifying factor. The results after normalization of ratings to CAGexp values between 40 to 44 in pre-HD AHT and in normotensive patients indicate that the pre-HD AHT patients show motor symptoms between 5 and 8 years later than normotensive patients with the same CAGexp, suggesting that AHT may be an AO modifier. This effect is remarkably longer than those reported for any genetic modifier in HD [9].

The mechanism underlying this observation is presently unknown. In fact, although AHT has been related with other neurodegenerative diseases, its effect is presently unclear. AHT has been associated with a reduced risk of Parkinson’s disease [6], but has also been considered a risk factor [7]. In AD, the relationship is also controversial [2], but most of the reviewed literature has noted that AHT is a strong risk factor [8], while antihypertensive drugs reduce the risk of AD [8,10]. Interestingly, most pre-HD AHT patients included in this study had started treatment with antihypertensive drugs prior to the onset of HD. Antihypertensive drugs have been suggested to confer a neuroprotective effect not only in AD [8,10], but also in PD [11], and they might also play a role in the later mAO shown by HD patients in our study.

The mechanism underlying this observation is presently unknown. In fact, although AHT has been related with other neurodegenerative diseases, its effect is presently unclear. AHT has been associated with a reduced risk of Parkinson’s disease [6] but has also been considered a risk factor [7]. In AD, the relationship is also controversial [2], but most of the reviewed literature has noted that AHT is a strong risk factor [8] while antihypertensive drugs reduce the risk of AD [8,10]. Interestingly, most pre-HD AHT patients included in this study had started treatment with antihypertensive drugs prior to the onset of HD. Antihypertensive drugs have been suggested to confer a neuroprotective effect, not only in AD [8,10], but also in PD [11], and they might also play a role in the later mAO shown by HD patients in our study.

Unfortunately, the high variability in the antihypertensive treatment types, changes in medication and incomplete medical records of our HD patient cohort have hampered our understanding of the impact of antihypertensive drugs in HD mAO. An additional limitation is that the prevalence of AHT increases with age [12], which means that a later onset HD patient is more likely to be recorded with a history of AHT, than an earlier onset participant. We have observed that the age of the participant at the time of data collection is correlated with mAO, as well as, with AHT. Thus, although age does not explain the mAO variability, it could have an influence in the observed relationship between mAO and AHT, leading to a spureous association. On the other hand, the mAO and AHT onset data suggest that the pre-HD AHT condition is not necessarily related to later onset ages: 1) the median age of AHT onset in pre-HD AHT cases is 45.5 years, which is similar to the mAO median in normotensives patients (45 years); 2) the majority of pre-HD AHT patients (71%) showed motor symptoms at < 60 years and there were no pre-HD AHT cases with late-onset mAO (>70 years); 3) the effect of AHT in mAO is detected in ranges of CAG (40–44) in which juvenil and late HD cases (extreme cases that could bias the result) are not manifested. Notwithstanding this evidence, we cannot rule out the possible influence that age of patients at the time of data collection could have on the pre-HD AHT condition and, consequently, on the observed association.

In spite of the caveats, given the lack of current treatment options for HD, we consider that the findings of our study merits to be reported considering the accessibility to AHT treatments and the potential impact of these drugs (or the AHT condition) in the onset of HD. We believe that the role of AHT and/or its treatment should be further examined in other HD cohorts such as ENROLL-HD and possibly in pre-clinical models.

## Supporting information

S1 Dataset 630 European adult-onset HD patient dataset. Age: patient’s age at the time of data collection; Sex: M (male) and F (female); CAGexp: CAG number repeat in expanded allele; mAO: motor onset, age of the first motor symptoms; AHT condition: normotensive (patient without arterial hypertension record), with AHT (patient with arterial hypertension records) and pre-HD AHT (patient with arterial hypertension records who manifested hypertension before HD symptoms).
(PDF)
