# Supplementary material for: Does arterial hypertension influence the onset of Huntington's disease?
Source: PLoS One. 2018 May 23;13(5):e0197975. doi: 10.1371/journal.pone.0197975 (PMC5965871; doi:10.1371/journal.pone.0197975)
Supplement: S1 Dataset — Age: patient’s age at the time of data collection; Sex: M (male) and F (female); CAGexp: CAG number repeat in expanded allele; mAO: motor onset, age of the first motor symptoms; AHT condition: normotensive (patient without arterial hypertension record), with AHT (patient with arterial hypertension records) and pre-HD AHT (patient with arterial hypertension records who manifested hypertension before HD symptoms). (PDF) [file pone.0197975.s001.pdf]

S1 Table. 630 European adult-onset HD patient dataset. Age: patient's age at the time of data collection; Sex: M (male) and F (female); CAGexp: CAG number repeat in expanded allele; mAO: motor onset, age of the first motor symptoms; AHT condition: normotensive (patient without arterial hypertension record), with AHT (patient with arterial hypertension records) and pre-HD AHT (patient with arterial hypertension records who manifested hypertension before HD symptoms).

Patient	Age	Sex	CAGexp	mAO	AHT condition
1	54	M	43	50	Normotensive
2	42	M	47	36	Normotensive
3	63	M	41	57	Normotensive
4	55	F	43	48	Normotensive
5	32	M	47	28	Normotensive
6	67	M	41	58	Normotensive
7	60	F	43	54	Normotensive
8	50	F	43	49	Normotensive
9	52	F	44	32	Normotensive
10	59	F	42	59	With AHT
11	69	M	41	61	Normotensive
12	59	M	42	56	Normotensive
13	26	M	50	22	Normotensive
14	40	M	45	34	Normotensive
15	62	F	41	60	Normotensive
16	48	F	43	47	Normotensive
17	44	F	46	32	Normotensive
18	46	M	45	38	Normotensive
19	43	M	47	36	Normotensive
20	45	F	46	27	Normotensive
21	35	F	49	28	Normotensive
22	55	F	45	41	Normotensive
23	69	F	41	67	Normotensive
24	32	M	47	24	Normotensive
25	45	M	43	43	Normotensive
26	47	F	44	42	Normotensive
27	68	M	40	57	Normotensive
28	54	F	41	53	Normotensive
29	38	M	46	30	Normotensive
30	40	M	46	32	Normotensive
31	57	F	47	35	Normotensive
32	60	M	43	50	Normotensive
33	54	M	43	43	Normotensive
34	46	M	47	44	Normotensive
35	45	F	47	35	Normotensive
36	63	M	43	59	Pre-HD AHT
37	56	F	43	48	Normotensive
38	57	M	42	51	Normotensive

39	28	F	50	28	Normotensive
40	72	F	43	55	Normotensive
41	39	M	44	24	Normotensive
42	44	F	45	41	Normotensive
43	52	F	44	39	Normotensive
44	55	F	42	46	Normotensive
45	38	F	44	30	Normotensive
46	61	M	40	57	Normotensive
47	36	M	48	32	Normotensive
48	57	M	43	49	With AHT
49	67	M	41	61	Normotensive
50	59	F	42	51	Normotensive
51	62	F	42	54	Normotensive
52	38	M	43	41	Normotensive
53	42	F	45	36	Normotensive
54	77	F	40	65	Normotensive
55	42	M	45	37	Normotensive
56	73	M	40	63	Normotensive
57	48	F	42	31	Normotensive
58	62	F	40	59	Normotensive
59	42	M	48	26	Normotensive
60	52	M	43	46	Normotensive
61	40	F	47	35	Normotensive
62	43	F	49	35	Normotensive
63	38	M	50	36	With AHT
64	56	M	45	47	Normotensive
65	76	F	41	55	Normotensive
66	60	F	43	47	Normotensive
67	61	M	42	44	With AHT
68	55	F	41	52	Normotensive
69	33	M	49	25	Normotensive
70	37	M	46	34	Normotensive
71	77	F	41	68	Normotensive
72	50	F	42	49	Normotensive
73	67	F	43	56	Normotensive
74	39	F	48	31	Normotensive
75	65	F	41	57	Normotensive
76	57	F	42	49	Normotensive
77	40	F	47	39	Normotensive
78	49	M	43	45	Normotensive
79	41	F	47	32	Normotensive
80	54	M	42	51	Normotensive
81	53	F	43	50	Normotensive
82	56	M	44	41	Normotensive
83	63	M	41	52	Normotensive
84	60	F	40	49	Normotensive
85	48	M	43	45	Pre-HD AHT
86	64	M	42	44	Normotensive

87	67	F	42	63	Normotensive
88	42	M	46	35	Normotensive
89	34	F	50	21	Normotensive
90	39	F	46	34	Normotensive
91	52	F	40	48	Normotensive
92	48	F	43	46	Normotensive
93	40	M	46	37	Normotensive
94	40	F	46	37	Normotensive
95	63	M	40	52	Normotensive
96	36	F	46	34	Normotensive
97	40	F	45	31	Normotensive
98	47	M	46	38	Normotensive
99	41	M	47	28	Normotensive
100	58	M	42	56	Normotensive
101	50	F	45	49	Normotensive
102	44	M	47	38	Normotensive
103	46	M	46	41	Normotensive
104	54	M	44	41	Normotensive
105	48	M	43	47	Normotensive
106	52	F	45	47	Normotensive
107	61	M	42	57	Normotensive
108	58	F	43	53	Normotensive
109	54	M	41	48	Normotensive
110	63	F	43	57	Normotensive
111	52	M	42	46	Normotensive
112	82	M	40	70	Pre-HD AHT
113	59	F	40	55	Normotensive
114	45	M	44	41	Normotensive
115	72	M	43	54	Normotensive
116	67	F	42	60	Normotensive
117	45	M	45	43	With AHT
118	36	M	45	35	Normotensive
119	49	F	45	41	Normotensive
120	52	F	40	42	Normotensive
121	76	M	42	55	Normotensive
122	65	M	45	60	Normotensive
123	69	M	40	66	Normotensive
124	36	M	50	32	Normotensive
125	48	F	45	22	Normotensive
126	66	F	42	60	Normotensive
127	43	F	44	43	Pre-HD AHT
128	47	F	40	46	Normotensive
129	61	M	41	60	Normotensive
130	61	F	41	60	Normotensive
131	70	F	40	64	Pre-HD AHT
132	74	M	40	68	Normotensive
133	49	F	44	41	Normotensive
134	62	F	43	53	Normotensive

135	55	F	44	47	With AHT
136	54	F	43	52	Normotensive
137	47	F	44	43	Normotensive
138	39	F	44	32	Normotensive
139	44	F	46	38	Normotensive
140	31	M	48	31	Normotensive
141	68	M	43	54	Normotensive
142	47	M	44	37	Normotensive
143	31	F	50	35	With AHT
144	46	M	45	42	Normotensive
145	58	F	41	56	Pre-HD AHT
146	42	F	43	39	Normotensive
147	72	M	41	62	Normotensive
148	62	M	42	58	Normotensive
149	58	F	44	47	Normotensive
150	34	M	46	32	Normotensive
151	43	M	46	32	Normotensive
152	61	F	42	53	Normotensive
153	64	M	41	61	Normotensive
154	63	M	44	52	Normotensive
155	34	M	48	31	Normotensive
156	49	F	44	47	Normotensive
157	62	F	42	53	Normotensive
158	51	F	44	48	Normotensive
159	39	F	43	42	Normotensive
160	44	M	44	38	Normotensive
161	44	F	48	37	Normotensive
162	57	F	43	37	With AHT
163	64	F	42	56	Normotensive
164	52	F	44	40	Normotensive
165	44	M	43	39	Normotensive
166	61	M	42	44	Normotensive
167	63	M	42	45	Normotensive
168	45	F	44	37	Normotensive
169	42	M	48	37	Normotensive
170	35	F	45	34	Normotensive
171	72	F	42	63	Normotensive
172	32	F	48	28	Normotensive
173	67	F	40	60	Normotensive
174	59	F	43	48	Normotensive
175	54	M	42	40	With AHT
176	41	M	47	45	Normotensive
177	64	F	42	48	With AHT
178	42	M	45	38	Normotensive
179	63	F	42	60	Pre-HD AHT
180	35	M	44	38	Normotensive
181	57	M	44	48	Normotensive
182	45	F	48	34	Normotensive

183	43	F	47	34	Normotensive
184	45	F	44	39	Normotensive
185	57	M	44	43	Normotensive
186	61	M	41	57	Normotensive
187	58	F	45	56	Normotensive
188	44	F	45	36	Normotensive
189	48	F	43	48	Normotensive
190	69	M	42	61	With AHT
191	75	F	40	66	Normotensive
192	60	M	40	57	Normotensive
193	40	F	46	37	Normotensive
194	66	M	41	65	Normotensive
195	43	M	47	34	Normotensive
196	41	M	45	35	Normotensive
197	42	F	44	38	Normotensive
198	28	M	48	26	Normotensive
199	41	M	45	37	With AHT
200	27	M	49	25	Normotensive
201	41	M	46	37	Normotensive
202	44	M	44	42	Normotensive
203	39	F	48	32	Normotensive
204	55	M	43	47	Normotensive
205	63	F	43	50	With AHT
206	40	F	48	32	Normotensive
207	69	M	41	67	Pre-HD AHT
208	53	M	43	48	Normotensive
209	48	F	44	42	Normotensive
210	62	F	43	41	Normotensive
211	47	M	45	47	Normotensive
212	56	F	43	45	Normotensive
213	29	F	46	35	With AHT
214	37	F	47	34	Normotensive
215	36	M	47	36	Normotensive
216	53	M	42	48	Normotensive
217	50	M	47	44	Normotensive
218	39	M	44	38	Normotensive
219	54	F	43	48	Normotensive
220	53	M	43	49	Normotensive
221	57	M	42	54	Pre-HD AHT
222	51	M	44	46	Normotensive
223	66	F	41	61	Normotensive
224	59	F	42	57	Normotensive
225	63	F	42	61	Normotensive
226	48	F	47	32	Normotensive
227	49	M	40	49	Normotensive
228	53	M	42	47	Normotensive
229	39	F	48	31	Normotensive
230	58	F	43	57	Pre-HD AHT

231	51	F	41	52	Normotensive
232	58	M	42	53	Normotensive
233	69	M	41	57	Pre-HD AHT
234	47	M	44	32	Normotensive
235	26	M	49	28	Normotensive
236	50	F	48	41	With AHT
237	43	M	45	41	With AHT
238	40	M	47	37	Normotensive
239	72	M	41	68	Normotensive
240	58	M	44	57	Normotensive
241	65	F	42	61	Normotensive
242	63	M	42	53	With AHT
243	49	F	43	47	Normotensive
244	52	F	42	41	Normotensive
245	81	F	40	75	Normotensive
246	53	F	43	52	Normotensive
247	43	M	44	37	Normotensive
248	47	F	44	41	Normotensive
249	47	F	42	46	Normotensive
250	40	M	47	38	Normotensive
251	58	F	42	49	Normotensive
252	64	M	42	61	Normotensive
253	54	M	43	49	Normotensive
254	41	M	43	43	Normotensive
255	40	M	45	41	Normotensive
256	62	F	42	49	Normotensive
257	55	F	42	44	Normotensive
258	65	F	40	65	Normotensive
259	51	M	46	48	Normotensive
260	53	M	42	49	Normotensive
261	52	F	44	40	Normotensive
262	55	M	44	50	Normotensive
263	66	F	43	61	Normotensive
264	43	M	43	44	Normotensive
265	65	F	43	59	Normotensive
266	46	F	46	44	Normotensive
267	74	M	40	55	With AHT
268	34	F	49	32	Normotensive
269	36	M	48	36	Normotensive
270	47	F	44	43	Normotensive
271	52	F	40	45	With AHT
272	56	M	42	51	Normotensive
273	47	F	44	38	Normotensive
274	38	M	47	35	Normotensive
275	39	M	46	34	Normotensive
276	42	M	46	38	Normotensive
277	69	F	40	59	Normotensive
278	73	M	41	68	Normotensive

279	69	F	41	61	With AHT
280	49	F	45	39	Normotensive
281	39	F	47	38	Normotensive
282	54	F	45	35	Normotensive
283	34	F	48	34	Normotensive
284	51	F	44	46	Normotensive
285	57	M	45	40	With AHT
286	57	M	43	51	Normotensive
287	55	F	41	53	Normotensive
288	46	M	40	36	Normotensive
289	49	F	44	46	Normotensive
290	38	F	47	31	Normotensive
291	65	F	41	58	Normotensive
292	44	M	45	44	Normotensive
293	61	M	42	53	With AHT
294	61	M	43	55	Pre-HD AHT
295	45	F	42	41	Normotensive
296	40	M	41	43	Normotensive
297	61	M	42	57	Pre-HD AHT
298	63	F	42	55	Normotensive
299	64	F	41	62	Normotensive
300	52	M	44	44	Normotensive
301	37	M	46	35	Normotensive
302	55	M	41	47	With AHT
303	66	F	41	58	Pre-HD AHT
304	36	M	48	31	Normotensive
305	47	M	45	30	Normotensive
306	50	M	48	30	Normotensive
307	51	M	44	51	With AHT
308	48	M	44	47	Normotensive
309	49	M	44	48	Normotensive
310	33	M	49	31	Normotensive
311	57	M	41	52	Normotensive
312	68	M	41	57	Normotensive
313	58	F	43	48	Normotensive
314	42	M	47	39	Normotensive
315	49	M	43	46	With AHT
316	36	F	50	32	Normotensive
317	68	F	44	66	Normotensive
318	41	F	46	39	Normotensive
319	55	F	42	52	Normotensive
320	66	M	42	55	Normotensive
321	48	F	45	40	With AHT
322	57	F	42	57	Normotensive
323	58	F	42	48	Normotensive
324	65	M	41	49	Normotensive
325	36	M	48	36	Normotensive
326	41	F	45	39	Normotensive

327	74	M	41	64	Normotensive
328	48	F	48	46	With AHT
329	30	M	50	28	Pre-HD AHT
330	51	F	44	41	Normotensive
331	55	F	42	50	Normotensive
332	43	M	46	33	Normotensive
333	36	F	49	30	Normotensive
334	49	F	41	45	Normotensive
335	45	F	45	37	Normotensive
336	45	F	43	45	Normotensive
337	53	F	44	40	Normotensive
338	45	M	45	43	Normotensive
339	58	M	42	54	Normotensive
340	56	F	40	54	Normotensive
341	68	F	41	64	Normotensive
342	35	F	46	39	Normotensive
343	46	M	45	42	Normotensive
344	42	M	47	34	Normotensive
345	40	M	47	32	Normotensive
346	44	F	44	39	Normotensive
347	46	M	44	41	Normotensive
348	60	F	41	37	Normotensive
349	59	F	41	51	Normotensive
350	63	M	43	54	Pre-HD AHT
351	45	F	46	45	Normotensive
352	48	F	42	44	Normotensive
353	33	M	47	28	Normotensive
354	42	F	46	38	Normotensive
355	50	M	46	35	Normotensive
356	63	M	43	49	Normotensive
357	52	F	41	51	Normotensive
358	66	M	45	56	Normotensive
359	65	F	42	47	Normotensive
360	57	M	42	30	Pre-HD AHT
361	69	M	42	63	Normotensive
362	35	F	50	35	Normotensive
363	46	M	42	46	Normotensive
364	44	M	44	43	Normotensive
365	59	M	43	50	Normotensive
366	50	M	44	40	Normotensive
367	56	M	42	50	Normotensive
368	66	F	47	63	Normotensive
369	67	M	42	58	Normotensive
370	52	M	43	50	Normotensive
371	47	M	45	44	Normotensive
372	58	F	43	43	With AHT
373	51	M	41	49	Pre-HD AHT
374	66	F	40	58	With AHT

375	48	M	45	46	Normotensive
376	60	M	42	43	Normotensive
377	46	F	42	36	Normotensive
378	69	M	40	55	With AHT
379	44	F	44	38	Normotensive
380	54	M	42	48	With AHT
381	61	F	43	55	Normotensive
382	44	M	44	43	Normotensive
383	61	M	44	53	Normotensive
384	51	F	43	36	Normotensive
385	41	M	46	40	Normotensive
386	42	F	46	25	Normotensive
387	64	F	43	62	Normotensive
388	53	M	44	40	Normotensive
389	36	F	49	24	Normotensive
390	66	F	45	51	With AHT
391	60	M	43	58	With AHT
392	54	F	41	50	Normotensive
393	48	F	46	45	Normotensive
394	67	F	43	52	Normotensive
395	43	F	44	39	Normotensive
396	43	F	47	34	Normotensive
397	54	F	42	47	Normotensive
398	67	F	42	65	Normotensive
399	35	M	48	28	Normotensive
400	58	M	42	52	With AHT
401	61	F	41	55	With AHT
402	70	F	41	65	Normotensive
403	53	M	44	43	Normotensive
404	45	M	45	41	Normotensive
405	71	F	41	59	Normotensive
406	42	M	46	35	Normotensive
407	67	F	50	60	Normotensive
408	52	M	42	52	Normotensive
409	58	M	42	54	Normotensive
410	70	F	40	67	Normotensive
411	55	M	45	49	Normotensive
412	60	F	43	59	With AHT
413	72	M	41	66	With AHT
414	66	F	40	60	With AHT
415	26	M	49	25	Normotensive
416	47	F	44	46	Normotensive
417	63	M	41	54	With AHT
418	41	M	45	36	Normotensive
419	41	M	43	31	With AHT
420	65	M	42	58	Normotensive
421	72	M	40	70	Normotensive
422	56	M	43	48	With AHT

423	36	F	43	34	Normotensive
424	46	M	46	39	Normotensive
425	43	F	47	30	Normotensive
426	61	M	42	48	Normotensive
427	45	F	41	36	Normotensive
428	47	F	44	39	Normotensive
429	44	M	43	41	Normotensive
430	56	F	45	44	Normotensive
431	50	F	46	35	Normotensive
432	54	M	47	49	Normotensive
433	41	M	47	36	Normotensive
434	60	M	41	57	Normotensive
435	43	F	46	36	Normotensive
436	39	M	41	38	Normotensive
437	46	M	46	37	Normotensive
438	60	F	41	53	Pre-HD AHT
439	55	F	40	51	Normotensive
440	31	F	45	34	Normotensive
441	51	M	42	45	Normotensive
442	62	M	42	52	Normotensive
443	47	M	44	44	Normotensive
444	55	M	44	45	Normotensive
445	47	F	43	46	Normotensive
446	48	M	45	30	Normotensive
447	57	F	42	53	Normotensive
448	35	F	47	33	Normotensive
449	51	F	44	43	Normotensive
450	68	M	42	59	Normotensive
451	47	F	43	43	Normotensive
452	53	F	44	35	Normotensive
453	49	M	45	40	Normotensive
454	71	F	41	65	Normotensive
455	52	M	43	46	Normotensive
456	54	F	43	50	Normotensive
457	65	F	41	50	Normotensive
458	48	M	44	43	Normotensive
459	55	F	44	52	Pre-HD AHT
460	40	M	45	39	Normotensive
461	38	F	44	38	Normotensive
462	62	M	42	59	With AHT
463	42	M	47	40	Normotensive
464	57	F	42	55	Pre-HD AHT
465	41	M	45	39	With AHT
466	65	M	42	62	With AHT
467	59	M	43	54	Normotensive
468	53	M	43	48	Normotensive
469	55	F	42	48	Normotensive
470	62	F	42	58	Normotensive

471	59	M	44	57	Normotensive
472	58	M	44	50	Pre-HD AHT
473	73	M	40	73	Normotensive
474	76	F	40	64	Normotensive
475	58	F	42	53	With AHT
476	61	F	41	58	Normotensive
477	69	M	40	67	Normotensive
478	50	F	43	46	Normotensive
479	53	M	43	45	Normotensive
480	78	M	40	65	Pre-HD AHT
481	59	M	42	56	With AHT
482	74	M	40	67	Normotensive
483	35	M	49	31	Normotensive
484	51	F	45	48	With AHT
485	69	F	43	62	With AHT
486	58	M	42	57	Normotensive
487	40	M	44	39	Normotensive
488	38	F	46	29	Normotensive
489	68	M	41	65	Normotensive
490	60	M	42	53	Normotensive
491	69	F	41	66	Normotensive
492	46	F	49	31	Normotensive
493	51	M	43	39	Normotensive
494	63	F	40	55	Normotensive
495	49	F	45	47	Normotensive
496	54	F	43	54	Normotensive
497	61	M	42	49	Normotensive
498	68	M	41	61	Normotensive
499	52	M	44	40	Normotensive
500	60	M	42	50	Normotensive
501	60	F	43	57	Normotensive
502	82	M	41	71	Normotensive
503	45	F	43	47	Normotensive
504	41	M	48	34	Normotensive
505	63	F	41	60	Normotensive
506	36	M	50	32	Normotensive
507	49	M	45	38	Normotensive
508	60	M	43	57	Normotensive
509	46	M	44	37	With AHT
510	49	F	43	47	Normotensive
511	56	F	43	47	Normotensive
512	69	M	44	67	Normotensive
513	52	M	45	48	Normotensive
514	41	F	47	41	Normotensive
515	43	F	44	38	Normotensive
516	87	F	40	70	With AHT
517	48	M	43	49	Normotensive
518	35	M	49	33	Normotensive

519	67	M	40	65	Normotensive
520	67	M	41	59	Normotensive
521	59	F	43	42	Normotensive
522	35	M	49	27	Normotensive
523	52	F	43	49	Normotensive
524	63	F	42	47	Normotensive
525	55	M	44	47	With AHT
526	69	M	42	68	Pre-HD AHT
527	59	F	41	54	Normotensive
528	49	M	42	41	Normotensive
529	58	F	45	53	Normotensive
530	74	F	40	61	Normotensive
531	55	F	43	47	Normotensive
532	57	F	42	51	Normotensive
533	52	F	44	51	Normotensive
534	39	M	47	39	Normotensive
535	27	F	47	25	Normotensive
536	54	F	44	49	Normotensive
537	52	M	42	43	Normotensive
538	40	M	48	39	Normotensive
539	51	F	44	41	Normotensive
540	56	M	41	55	With AHT
541	61	F	41	58	Normotensive
542	54	M	43	54	Normotensive
543	40	M	47	36	Normotensive
544	66	F	42	54	Normotensive
545	55	F	42	51	Normotensive
546	56	F	43	46	Normotensive
547	65	F	40	58	Normotensive
548	79	M	40	59	Normotensive
549	32	F	47	30	Normotensive
550	40	F	45	38	Normotensive
551	60	F	40	55	With AHT
552	67	F	41	63	Normotensive
553	54	M	43	53	Normotensive
554	56	M	42	53	Normotensive
555	47	F	46	43	Normotensive
556	54	F	45	44	Normotensive
557	64	M	42	57	Normotensive
558	64	F	41	60	Pre-HD AHT
559	47	M	42	35	Normotensive
560	53	M	44	43	Normotensive
561	59	F	42	57	Normotensive
562	58	F	43	53	Normotensive
563	35	F	47	35	Normotensive
564	50	F	44	45	Normotensive
565	48	M	45	48	Normotensive
566	58	M	40	51	Normotensive

567	37	F	46	30	Normotensive
568	53	F	43	43	With AHT
569	50	M	44	42	Normotensive
570	29	F	48	21	Normotensive
571	57	M	43	53	Normotensive
572	52	F	43	38	Normotensive
573	43	M	46	41	Normotensive
574	49	F	40	49	Normotensive
575	45	F	46	40	Normotensive
576	51	F	43	42	Normotensive
577	63	M	40	49	Normotensive
578	57	F	44	55	Normotensive
579	32	M	49	30	Normotensive
580	57	F	43	56	Normotensive
581	67	M	40	63	With AHT
582	44	M	44	42	Normotensive
583	40	F	47	33	Normotensive
584	34	M	46	24	Normotensive
585	62	M	42	56	With AHT
586	55	M	42	45	Normotensive
587	38	F	43	36	Normotensive
588	62	M	42	59	Normotensive
589	48	F	46	40	Normotensive
590	67	M	42	57	Normotensive
591	56	M	43	50	Normotensive
592	60	F	41	61	Pre-HD AHT
593	60	M	42	53	Normotensive
594	57	M	42	48	Normotensive
595	50	F	41	44	Normotensive
596	53	M	44	38	Normotensive
597	52	M	44	45	Normotensive
598	58	M	41	42	With AHT
599	52	M	44	47	Normotensive
600	42	M	45	42	Normotensive
601	35	M	47	34	Normotensive
602	56	F	43	46	Normotensive
603	61	F	40	52	Normotensive
604	67	F	44	64	Normotensive
605	54	F	41	56	Normotensive
606	59	F	43	58	Normotensive
607	57	F	42	52	Normotensive
608	36	F	47	35	Normotensive
609	56	F	42	50	Normotensive
610	66	F	40	63	Normotensive
611	56	F	42	55	Normotensive
612	41	M	50	32	Normotensive
613	46	M	44	43	Normotensive
614	51	M	46	49	Normotensive

615	55	M	42	46	Normotensive
616	59	F	43	44	Normotensive
617	38	F	47	30	Normotensive
618	52	F	43	47	Pre-HD AHT
619	56	M	40	47	Normotensive
620	48	M	44	42	Normotensive
621	53	M	43	46	With AHT
622	53	F	41	50	Pre-HD AHT
623	53	M	42	40	Normotensive
624	44	M	49	39	Normotensive
625	49	M	42	44	Normotensive
626	30	M	48	25	Normotensive
627	43	F	47	32	Normotensive
628	53	F	42	53	Normotensive
629	73	F	41	61	Normotensive
630	64	F	41	57	Normotensive
